# Metformin: From medieval age to new therapeutic targets

**DOI:** 10.4103/0973-3930.41984

**Published:** 2008

**Authors:** Alexandre Leite de Souza

**Affiliations:** Intensive Care Unit, Emílio Ribas Institute of Infectology, São Paulo, Brazil

Dear Sir,

The recent review by Tandon[1] of metformin therapy describes the new uses of this widely prescribed biguanide in the United States. I would like to draw attention to several points made in this review.

The author has stated that, ‘the effects beyond the conventional hypoglycemic effects of metformin offer advantages over other available oral hypoglycemic agents.’ However, it should be noted that metformin lowers the blood glucose concentration without causing hypoglycemia. Unlike the sulfonylureas, metformin does not produce hypoglycemia in either patients with type 2 diabetes or in normal subjects and it does not increase pancreatic release of insulin. In fact, it decreases plasma glucose levels by stimulation of peripheral glucose uptake by muscle and adipose tissue.[2] In adipose tissue and skeletal muscle, metformin, in the presence of insulin, facilitates the trafficking of the glucose transporters 1 and 4 to the plasma membrane.[2] Therefore, metformin is an anti-hyperglycemic agent and not a hypoglycemic agent.

The author also states that ‘polycystic ovary syndrome (PCOS) thus resembles the metabolic syndrome.’ In fact, the metabolic syndrome is a critical pathway of the PCOS in most affected women.[3] Therefore, the metabolic syndrome is directly linked to the PCOS pathogenesis.

Another statement made by the author with regard to metformin is: ‘…in the absence of insulin resistance or diabetes, it cannot be used as weight-loss agent.’ The prevalence of adult obesity in the United States has more than doubled from 15% in the late 1970s to 31% in 2000.[4] Among adolescents the prevalence has tripled from 5% to 15% over the same period.[4] Faced with this dramatic epidemic of obesity and on the basis of some studies,[2] metformin therapy might be considered a potential agent for the prevention of diabetes in those individuals who are overweight and have mild hyperglycemia, principally in younger patients who have failed initial attempts at lifestyle modification.

Historically, in the medieval period, *Galega officinalis* was found to reduce the extreme diuresis associated with diabetes mellitus. The key element was subsequently found to be a guanidine.[2] Deciphering the pathogenesis of diabetes, cardiovascular phenomena,[5] and the metabolic syndrome could expand our knowledge and lead to new therapeutic targets.
